# A154 PRIMARY APPENDICEAL GASTROINTESTINAL STROMAL TUMOR (GIST) IN A PATIENT WITH DELAYED APPENDICITIS: CASE PRESENTATION AND LITERATURE REVIEW

**DOI:** 10.1093/jcag/gwad061.154

**Published:** 2024-02-14

**Authors:** I I Pacheco Blandino, K Naser

**Affiliations:** Western University, London, ON, Canada; Queen's University, Kingston, ON, Canada

## Abstract

**Background:**

Gastrointestinal stromal tumors (GISTs) are uncommon neoplasias, arising from mesenchymal tissue in the gastrointestinal tract. However, primary appendiceal GISTs (PA-GISTs) are rare, with less than 27 cases reported to date. Although treatable with neoadjuvant chemotherapy and subsequent resection, GISTs of the appendix can initially present with appendicitis-like symptoms, and are often treated as acute appendicitis (AA) due to initial misdiagnosis. A 75-year-old man presented to the emergency department of Brightshores Health System (BSHS) with right upper abdominal pain progressing to the right lower quadrant. He was referred to our surgical service, and his condition was treated as delayed appendicitis.

**Aims:**

1. To raise awareness of GIST as a distinct diagnostic possibility in patients presenting with acute appendicitis (AA)-like symptoms.

2. To assess whether delayed appendectomy has an impact in the overall disease outcome for PA-GIST.

**Methods:**

A rare case of PA-GIST was found after a retrospective review of all appendectomies performed at BSHS for appendicitis with or without neoplasia in the last 11 years. To our surprise, we uncovered a rare case PA-GIST. Comprehensive chart review was performed.

**Results:**

A 75-year-old man presenting with a 5-day history of right upper and lower abdominal pain was clinically diagnosed with AA. CT abdomen and ultrasound showed probable appendicitis with phlegmon, distal tip inflammation, no abcess and mild left hydronephrosis. After initial antibiotics and colonoscopy, the patient underwent interval appendectomy. Pathology revealed no AA, a low-grade GIST with focal interstitial cell of Cajal hyperplasia of the distal appendix and positive margin. Six weeks later, cecectomy showed no clinicopathologic evidence of residual PA-GIST. Literature review revealed 27 other PA-GIST tumors to date. Twenty three were found incidentally through imaging, colonoscopy, or surgery. Four cases involved patients presenting with AA symptoms. Our patient was among those presenting with AA symptoms, but with no histological evidence of appendicitis in the delayed appendectomy specimen. Consistent with 25/27 cases, our patient’s GIST displayed spindle morphology, ampersand:003C5cm in size, and without atypia, mitosis, or necrosis.

**Conclusions:**

PA-GISTs tumors are rare, with only 28 cases reported to date, including ours. Some PA-GIST tumors are found incidentally in patients that present with symptoms of AA. Although there is no concensus opinion for optimal treatment of PA-GIST, delayed appendectomy followed by cecectomy have proven to be an acceptable treatment for our patient with no evidence of recurrent or metastatic disease after 6.5 years on follow up by clinical and endoscopic surveilance.

**Funding Agencies:**

None

